# A Case of Critically Ill Infant of Coronavirus Disease 2019 With Persistent Reduction of T Lymphocytes

**DOI:** 10.1097/INF.0000000000002720

**Published:** 2020-06-05

**Authors:** Liru Qiu, Rong Jiao, Aiming Zhang, Xi Chen, Qin Ning, Feng Fang, Fang Zeng, Niannian Tian, Yi Zhang, Yafei Huang, Ziyan Sun, Menaka Dhuromsingh, Hao Li, Yang Li, Rongrong Xu, Yu Chen, Xiaoping Luo

**Affiliations:** From the * Department of Pediatrics, Tongji Hospital, Tongji Medical College, Huazhong University of Science and Technology, Wuhan, Hubei, China; † Department of Pediatrics, Xiangyang No.1 People’s Hospital, Hubei University of Medicine, Xiangyang, Hubei, China; ‡ Department of Infectious Disease, Tongji Hospital of Tongji Medical College, Huazhong University of Science and Technology, Wuhan, Hubei, China; § Department of Public Management, Xiangyang No.1 People’s Hospital, Hubei University of Medicine, Xiangyang, Hubei, China; ¶ Department of Pathogen Biology, School of Basic Medicine, Tongji Medical College, Huazhong University of Science and Technology Wuhan, Hubei, China; ‖ Department of Radiology, Tongji Hospital, Tongji Medical College, Huazhong University of Science and Technology, Wuhan, Hubei, China; ** Division of Cardiology, Department of Internal Medicine, Tongji Hospital, Tongji Medical College, Huazhong University of Science and Technology, Wuhan, Hubei, China; †† Department of Laboratory, Xiangyang No.1 People’s Hospital, Hubei University of Medicine, Xiangyang, Hubei, China; ‡‡ Department of Radiology, Xiangyang No.1 People’s Hospital, Hubei University of Medicine, Xiangyang, Hubei, China.

**Keywords:** coronavirus disease 2019, infant, critical case, lymphopenia, T cells

## Abstract

The outbreak of coronavirus disease 2019 (COVID-19) is becoming a global threat. However, our understanding of the clinical characteristics and treatment of critically ill pediatric patients and their ability of transmitting the coronavirus that causes COVID-19 still remains inadequate because only a handful pediatric cases of COVID-19 have been reported.

**Methods:**

Epidemiology, clinical characteristics, treatment, laboratory data and follow-up information and the treatment of critically ill infant were recorded.

**Results:**

The infant had life-threatening clinical features including high fever, septic shock, recurrent apnea, petechiae and acute kidney injury and persistent declined CD3+, CD4+ and CD8+ T cells. The duration of nasopharyngeal virus shedding lasted for 49 days even with the administration of lopinavir/ritonavir for 8 days. The CD3+, CD4+ and CD8+ T cells was partially recovered 68 days post onset of the disease. Accumulating of effector memory CD4+ T cells (CD4+TEM) was observed among T-cell compartment. The nucleic acid tests and serum antibody for the severe acute respiratory syndrome coronavirus 2 of the infant’s mother who kept intimate contact with the infant were negative despite no strict personal protection.

**Conclusions:**

The persistent reduction of CD4+ and CD8+ T cells was the typical feature of critically ill infant with COVID-19. CD4+ and CD8+ T cells might play a key role in aggravating COVID-19 and predicts a more critical course in children. The prolonged nasopharyngeal virus shedding was related with the severity of respiratory injury. The transmission of SARS-CoV-2 from infant (even very critical cases) to adult might be unlikely.

## Abstract 2

Supplemental Digital Content is available in the text.

The current outbreak of the coronavirus disease 2019 (COVID-19) from the epicenter Wuhan city in Hubei Province has spread to numerous countries. Many publications have described the clinical and the laboratory characteristics of the critically ill adult patients.^1–3^ However, our understanding of the spectrum of pediatric cases, treatment of critically ill patients and their ability of transmitting the coronavirus that causes COVID-19 still remains inadequate because very few pediatric cases of COVID-19 have been reported.

In this article, we describe the complete clinical course and follow-up data of a critically ill infant with COVID-19 presenting with life-threatening clinical features including high fever, septic shock, recurrent apnea, petechiae and acute kidney injury.

## CASE PRESENTATION

An 8-month-old male infant with poor growth and malnutrition started coughing on January 25, 2020, after a routine hospital visit for physical examination a week earlier. His mother denied the history of any direct contact with COVID-19 patients. His cough was aggravated on January 31, and he was subsequently hospitalized. He had medical history of neonatal cardiac surgery (atrial and ventricular septal defects and aortic stenosis repairs) and pneumonia twice during his early infancy (Table S1, Supplemental Digital Content 1, http://links.lww.com/INF/D942; Table S2, Supplemental Digital Content 1, http://links.lww.com/INF/D942).

On the first day of the admission (7th illness day), upon evaluation, dull heart sounds, mottled skin, cold fingers and petechiae were found. The infant had wheezing and recurrent apnea and was supplied oxygen delivered by nasal cannula at 2 L/min. He had body temperature of 38.3°C with recurrent apnea, and the percutaneous oxygen saturation dropped to 60%–70% on several occasions. Chest radiograph showed increased density, profusion and thickened lung texture before the endotracheal intubation, small spot-like and patchy fuzzy shadow. He was given ventilator-assisted breathing via endotracheal intubation. The image of chest radiograph was improved a little after endotracheal intubation (Figure S1, Supplemental Digital Content 1, http://links.lww.com/INF/D942).

His urine output was 0.4 mL/kg·h with gross hematuria, and his lowest blood pressure recorded was 85/37 mm Hg. The SARS-CoV-2 nucleic acid tests of the nasopharyngeal swab and rectal swab were positive, and the other viral respiratory pathogens tests were negative (Table S3, Supplemental Digital Content 1, http://links.lww.com/INF/D942). He was diagnosed with COVID-19, and his clinical condition was critical. Laboratory investigations showed that the levels of lymphocytes, white blood cells, CD3+, CD4+, CD8+ T cells and fibrinogen were below the normal range, and the level of lactate dehydrogenase was increased (Tables 1 and 2). C3 and C4 were in the normal range.

**TABLE 1.**
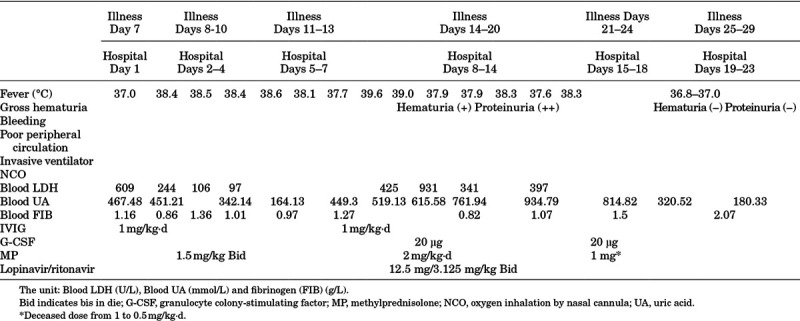
Treatment and Symptoms According to Day of Illness and Day of Hospitalization

**TABLE 2.**
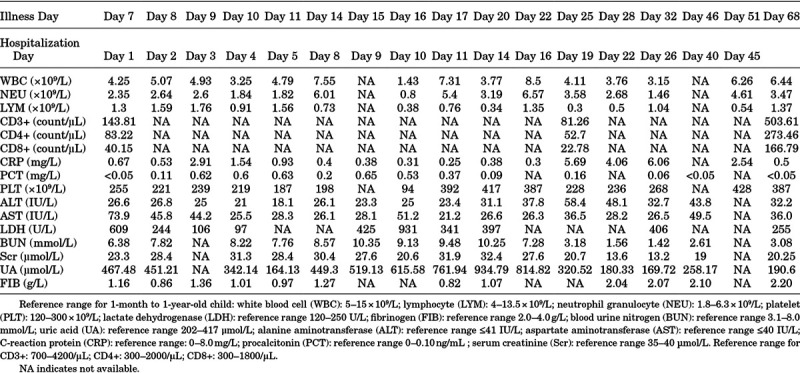
Clinical Laboratory Results During the Current Hospitalization

Initial treatment was the first dose of intravenous immune globulin (1 mg/kg·d, hospitalization days 1–2, illness days 7–8), methylprednisolone (1.5 mg/kg twice a day, hospitalization days 1–5, illness days 7–11) combined with the supportive therapy such as fluids and electrolytes, low-dose diuretics and dopamine to maintain blood pressure. Despite the above treatment, the patient still had fever (up to 38.6°C) and maintained a poor peripheral circulation. Lopinavir/ritonavir (12.5/3.125 mg/kg, twice daily) was introduced on the evening of hospitalization day 7 (illness day 13), followed by a second dose of intravenous immune globulin (1 mg/kg·d, hospitalization days 8–9, illness days 14–15), methylprednisolone (2 mg/kg·d, hospitalization days 8–14, illness days 14–20 days, tapering) (Table 1). The infant continued febrile on hospitalization days 8 and 9 with a body temperature of up to 39.6°C and a decrease in lymphocyte count to 0.38 × 10^9^/L. On the 4th and 10th days of hospitalization (10th and 16th illness days), 2 doses of granulocyte colony-stimulating factor were administered to improve the neutrophil count (decreased to 0.3 × 10^9^/L). The pyrexia started subsiding on hospitalization day 10 (day 3 of lopinavir/ritonavir administration), and an improvement blood pressure was observed. By the 15th day of hospitalization day (day 8 of lopinavir/ritonavir administration), he became afebrile and was off ventilator support.

**FIGURE 1.**
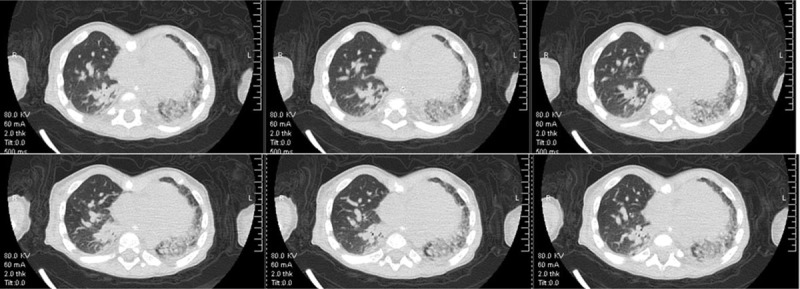
The computed tomographic (CT) scan on the 17th hospitalization day (illness day 23). Multiple ground-glass opacity and patchy and flaky high-density shadows in both lungs, enlargement of hilar shadows and thickened pulmonary vessels.

Chest computed tomographic scans showed multiple ground-glass opacity and patchy and flaky high-density shadows in both lungs (hospitalization day 17, illness day 23) (Fig. 1), which improved in the scans that followed (hospitalization days 33 and 43, illness days 39 and 49) (Figure S2, Supplemental Digital Content 1, http://links.lww.com/INF/D942; Figure S3, Supplemental Digital Content 1, http://links.lww.com/INF/D942).

The infant’s severe acute respiratory syndrome coronavirus 2 (SARS-CoV-2) nucleic acids tests of rectal and nasopharyngeal swabs turned negative on the 38th day and 49th day of illness, respectively.

Upon discharge on March 16 (46th hospitalization day, 52nd illness day), the lymphocytes count was 0.54 × 10^9^/L (reference range: 4–13.5 × 10^9^/L). Both IgM and IgG antibody for SARS-CoV-2 were positive (colloidal gold based).

On the 68th day of illness, the CD3+, CD4+ and CD8+ T-cell count of the infant were still below the reference range. The percentage of CD3+CD4+CCR7−CD45RA− lymphocytes was 53.13% (in CD4+ T cells) despite sustained reduction of CD3+, CD4+ and CD8+ lymphocytes (Table S4, Supplemental Digital Content 1, http://links.lww.com/INF/D942). The ratio of Th1/Th2 was 0.31 (reference range: 0.73–18.5).

## DISCUSSION

Because critically ill pediatric cases with COVID-19 are unusual, little is known about the characteristics in this age group with such severity of illness.

This infant had typical critical features including high fever, septic shock, recurrent apnea, gross hematuria, hypofibrinogenemia, lymphopenia and acute kidney injury. The initial clinical manifestation of his critical condition was septic shock, which is similar to adults.^3^ Adult data showed that elderly patients with a history of comorbidities were more likely to progress to severe disease,^4^ because those comorbidities associated with low-grade systemic inflammation, such as diabetes and hypertension.^5^ The defects in T-cell and B-cell function of elder patients could lead to a deficiency in control of viral replication and more prolonged proinflammatory responses, potentially leading to poor outcome.^6^ The medical history of cardiac surgery, pneumonia, failure to thrive and malnutrition of this infant might trigger the chronic inflammatory state and predisposing risk factors for the progression to critical stage. Therefore, more emphasis should be put on infants with comorbidities especially involved the immune system and immunodeficiency during the global epidemic phase of COVID-19.

Research about the clinical features of 8 severe pediatric patients with COVID-19 showed no significant decrease in peripheral CD3+, CD4+ and CD8+ T lymphocytes.^7^ It also has been observed in other noncritically ill (including severe cases) COVID-19 pediatric patients.^8,9^ However, this is completely different in our case. Significant decrease in CD3+, CD4+ and CD8+ lymphocytes was observed during hospitalization of this infant. The circulating lymphocytes, mainly CD3+, CD4+ and CD8+ T cells, dramatically declined and were sustained for at least 68 days. Data of adults also showed that lymphopenia and reduction of CD4+ and CD8+ T cells were the clinical features observed in severe COVID-19 patients.^1–4^ The reduction of T cells also was the typical feature of this critically ill infant. The lymphocytes subsets were not checked in this infant: however, the complete white test showed that the count of the lymphocytes was in the normal range when he was 6 months old (Table S2, Supplemental Digital Content 1, http://links.lww.com/INF/D942).

CD4+ T cells play an important role in immune system and orchestrate the deletion and amplification of immune cells. The depletion of CD4+ T cells leads to an enhanced immune-mediated interstitial pneumonitis and delayed clearance of SARS-CoV from the lungs.^10^ These data highlight that the reduction of CD4+ T cells might be related to disease severity of COVID-19, including in children.

Despite the reduction of CD4+ lymphocytes, an accumulation of effector memory CD4+ T cells (CD4+TEM) (53.13% in CD4+ lymphocytes, 0.145 × 10^9^/L) was found in this infant. This indicates the presence of memory T-cell response, and SARS-CoV-2 had not altered the capacity of accumulation of CD4+TEM.

The patient’s nasopharyngeal swab nucleic acid tests remained positive for 49 days and are the longest positive nasopharyngeal test duration reported in children with COVID-19, surpassing a reported surviving adult case where the nasopharyngeal swab was positive for COVID-19 up to 37 days.^3^ The analysis of 10 pediatric patients who had mild COVID-19 indicated that nasopharyngeal viral shedding was about 2–3 weeks.^9^ None, however, had severe lymphopenia. An adult cohort study showed the nasopharyngeal virus was continuously positive until death in nonsurvivors of COVID-19.^3^ The longer nasopharyngeal viral shedding in this infant may be related to the high nucleic acid load and the reduced ability of virus clearance after methylprednisolone use. The use of steroid also affects lymphocyte count. It remains unclear whether the persistent decline of T cells contributes to nasopharyngeal virus clearance or COVID-19 progression. Short-term, low-to-medium dose of glucocorticoids were recommended by the Chinese Pediatric Society of the Chinese Medical Association for management of severe and critical COVID-19 infection in children.^11^

This was the first pediatric COVID-19 patient underwent a treatment with lopinavir/ritonavir in China. Symptomatic remissions were observed after the administration of lopinavir/ritonavir, but increased uric acid levels were recorded. Serum uric acid increased to 934.79 mmol/L (day 7 of lopinavir/ritonavir administration). Three days after the discontinuation of lopinavir/ritonavir, the uric acid decreased to normal on the 19th hospitalization day (25th illness day). It is unknown whether the increased uric acid in this case was related to lopinavir/ritonavir or due to COVID-19. Lopinavir/ritonavir should be used with caution in pediatric COVID-19 and requires large-scale trials.

With respect to the SARS-CoV-2 pneumonia epidemic in Hubei Province, it is estimated that the infant was infected with the SARS-CoV-2 during the hospital visit on January 17, 2020. The infant had been living at home with his mother and sleeping together for the 14 days post infection. The infant had been taken care of by his mother. Neither of them wore a mask at home. There is a high probability that the infant got infected during the hospital visit with his mother because otherwise he had been at home. His mother’s nucleic acid tests and antibody tests were negative. This indicates that infant might be more susceptible to SARS-CoV-2 infection. This epidemiologic analysis suggests the unlikelihood of transmission of COVID-19 even from critically ill infant to adult.

## Supplementary Material


