# Supplementary material for: A Case of Critically Ill Infant of Coronavirus Disease 2019 With Persistent Reduction of T Lymphocytes
Source: Pediatr Infect Dis J. 2020 Jun 5;39(7):e87–90. doi: 10.1097/INF.0000000000002720 (PMC7279056; doi:10.1097/INF.0000000000002720)
Supplement: Supplementary file 1 [file inf-39-0e87-s001.docx]

**Supplementary Appendix**

**Table of contents**

| Diagnostic criteria | 2 |
| --- | --- |
| Method | 3 |
| Table S1. The clinical course of the infant | 6 |
| Table S2. White blood cells and lymphocyte at 6 months old | 7 |
| Table S3. The other viral respiratory pathogens tests | 8 |
| Table S4. Lymphocyte subsets of the infant on the 68th illness day | 9 |
| Figure S1. Chest X ray images | 11 |
| Figure S2. Chest CT scans images: hospitalization day 33 | 12 |
| Figure S2. Chest CT scans images: hospitalization day 43 | 13 |
| Author contributions | 14 |
| Funding | 14 |
| Acknowledgement | 14 |
| Declaration of interests | 15 |
| References | 15 |

**Diagnostic criteria:**

Shock^1^ was defined according to the International Pediatric Sepsis Consensus Conference published definitions and criteria for sepsis and septic shock

Acute kidney Injury (AKI) ^2^ was diagnosed according to the KDIGO clinical practice guidelines

Schwartz formula^3^:according the calculation of Schwartz formula

Critical case^4^: was defined according to the recommendation of Chinese Pediatric Society of the Chinese Medical Association

**Methods:**

Data collection:

The medical records of the infants were analyzed, including medical history, exposure history, and family life. A detailed analysis of patient records was performed. The date of disease onset was defined as the day when the cough was noticed by his parents.

Specimen Collection：

The nasal or anus swab specimen was collected from the infant. Swabs were placed immediately in 3mL viral transport media (VTM) and transported to the laboratory.

Testing

The nasal swab specimen was collected from the infant. Swabs were placed immediately in 3 mL viral transport media (VTM)

and transported to the laboratory. Total nucleic acid was extracted from 200μl of throat swab specimen, using fully automated Nucleic Acid Extraction System 9600E (Xi'an TianLong Science and Technology Co., Ltd., Xi’an, China). Diagnosis of SARS-CoV-2 infection was made using real-time RT–PCR in the laboratory. Two target genes were simultaneously amplified and tested, including open reading frame 1ab(ORF1ab) and nucleoprotein（N）^5^. The following primers were used for the detection of ORF1ab gene and N gene of SARS-CoV-2 (<http://ivdc.chinacdc.cn/kyjz/202001/t20200121_211337.html>.):

**ORF1ab gene**

| Forward primer | CCCTGTGGGTTTTACACTTAA |
| --- | --- |
| Reverse primer | ACGATTGTGCATCAGCTGA |
| The probe | 5'-FAM-CCGTCTGCGGTATGTGGAAAGGTTATGG-BHQ1-3' |

**N gene**

| Forward primer | GGGGAACTTCTCCTGCTAGAAT |
| --- | --- |
| Reverse primer | CAGACATTTTGCTCTCAAGCTG |
| The probe | 5'-FAM-TTGCTGCTGCTTGACAGATT-TAMRA-3' |

Diagnostic Testing for SARS-CoV-2:

These diagnostic criteria were based on the recommendation by the National institute for viral disease control and prevention of CDC (China) (http://ivdc.chinacdc.cn/kyjz/202001/t20200121_211337.html). Details are provided in the Supplementary Appendix.China Food and Drug Administration (CFDA)approved clinical diagnostic kit (DAAN Gene Co., Ltd) were applied. A Ct value ≤40 was defined as positive and confirmed as COVID-19 cases. A Ct value >40 was defined as negative.

CFDA approved clinical diagnostic kits were used for the detection of other potential respiratory viruses using real-time PCR, including influenza A virus, influenza B virus and respiratory syncytial virus, as well as mycoplasma pneumoniae, adenovirus, and avian influenza virus H7 subtypes.

Blood SARS-Cov-2 Antibody: CFDA approved clinical diagnostic kits of 2019-nCov antibody IgM&IgG (Vazyme Medical Technology Co.,LTD, Nanjin,China) Following the introductions of the Kit

**Ethic:**

All examinations and investigations in this case were approved by the Ethical Committee of Tongji Hospital of Huazhong University of Science & Technology (China) (TJ-C20200147)and were conducted in accordance with the Declaration of Helsinki. Written informed consent was obtained from the parents for publication of this case report and accompanying images.

**Table S1** **The clinical course of the infant**

**May,2019**

**January,2020**

| hospitalization | | | |  | | | | | hospitalization | |  | | | hospitalization | | | |  |
| --- | --- | --- | --- | --- | --- | --- | --- | --- | --- | --- | --- | --- | --- | --- | --- | --- | --- | --- |
|  | | | | **at home** | | | | |  | | **at home** | | |  | | | | |
|  | **invasive**  **ventilator** | | **cough**  **cough** | | | | | | | | | | | | **invasive ventilator** |  | | |
|  | | | | | | | | | | | | | | | | | | |
|  | **pneumonia** | | |  | | |  | **pneumonia** | | | |  | |  | **COVID-19 critically ill** | | | |
| 1month | 2months | 3 months | | 4months | 5months | 6months | | | | 7months | | 8months | 9months | | 10months | | 11months | |

**Birthday**

**Surgery***

**Discharged March 16,2020**

**Physical examination**

*Surgery: atrial and ventricular septal defect repairs and aortic stenosis of congenital cardiac defects

**Table S2** **white blood cells and lymphocyte at 6 months old**

|  | WBC(×10^9^/L) | NEU(×10^9^/L) | LYM(×10^9^/L) | PLT(×10^9^/L) | HGB（×g/L） |
| --- | --- | --- | --- | --- | --- |
| 6 months old | 7.28 | 2.8 | 3.32 | 490 | 127 |

Normal range for one month to 1-year-old child:

White blood cell count(WBC): 5-15 x 10^9^/L

Lymphocyte (LYM): 4-13.5x 10^9^/L

Neutrophile (NEU):1.8-6.3 x 10^9^/L

platelet (PLT): 120-300×10^9^/L PLT

**Table S3 The other viral respiratory pathogens tests**

| pathogens | results |
| --- | --- |
| (influenza A virus | negative |
| influenza B virus | negative |
| respiratory syncytial virus | negative |
| mycoplasma pneumoniae | negative |
| adenovirus | negative |
| avian influenza virus H7 subtypes | negative |

**Table S4 The results of lymphocyte subsets on 68^th^ illness day**

| Phenotype | Denominator of ratio | Result（%） |
| --- | --- | --- |
| CD34+CD45dim7-AAD- | WBC | 0.05 |
| CD45+SS(high) | WBC | 57.62 |
| CD45+SS(mid) | WBC | 19.56 |
| Lin-HLA-DR+ | WBC | 0.44 |
| Lin-HLA-DR+CD11c-CD123+ | WBC | 0.23 |
| Lin-HLA-DR+CD11c+ | WBC | 0.14 |
| CD45+SS(low)FS(low) | WBC | 21.11 |
| CD3-CD56+ | LYM | 22.76 |
| CD3+CD56+ | LYM | 0.55 |
| CD3+ | LYM | 36.76 |
| CD3+CD69+ | LYM | 0.61 |
| CD3+CD25+ | LYM | 3.16 |
| CD3+HLADR+ | LYM | 2.03 |
| CD3+CD4+ | T cells | 54.31 |
| CD3+CD4+CCR7+CD45RA+ | CD4+T cells | 27.68 |
| CD3+CD4+CCR7+CD45RA- | CD4+T cells | 16.84 |
| CD3+CD4+CCR7-CD45RA+ | CD4+T cells | 2.34 |
| CD3+CD4+CCR7-CD45RA- | CD4+T cells | 53.13 |
| CD3+CD4+CD25(high)CD127(dim) | CD4+T cells | 7.66 |
| CD3+CD8+ | T cells | 33.12 |
| CD3+CD8+CCR7+CD45RA+ | CD8+ T cells | 36.59 |
| CD3+CD8+CCR7+CD45RA- | CD8+ T cells | 5.1 |
| CD3+CD8+CCR7-CD45RA+ | CD8+ T cells | 3.81 |
| CD3+CD8+CCR7-CD45RA- | CD8+ T cells | 54.5 |
| CD3+CD4-CD8- | T cells | 11.13 |
| CD3+CD4+CD8+ | T cells | 1.45 |
| TCR-γδ CD3+ | T cells | 17.95 |
| TCR-γδ CD3+CD8+ | TCR-γδ T cells | 46.86 |
| CD19+CD3- | LYM | 35 |
| CD19+CD3-CD5+ | B cells | 6.77 |
| CD19+CD3-CD5- | B cells | 93.23 |

**Figure S1 X-ray before and after the endotracheal intubation (hospitalization day 1,2, illness day 7,8)**


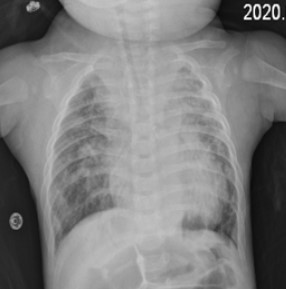

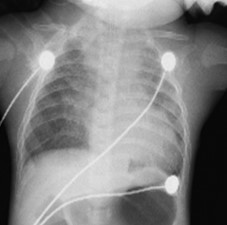


**B**

**A**

Chest X-ray before and after the endotracheal intubation (hospital day 1 and day 2)

A．Before the endotracheal intubation： Increased in density, profusion and thickened lung texture, small spot-like and patchy fuzzy shadow can be seen along the lung texture

B．After the endotracheal intubation： Both lung texture is clearer than A， sheet-like high density shadow

**Figure S2** **C****hest CT scans (hospitalization day 33, illness day 39)**


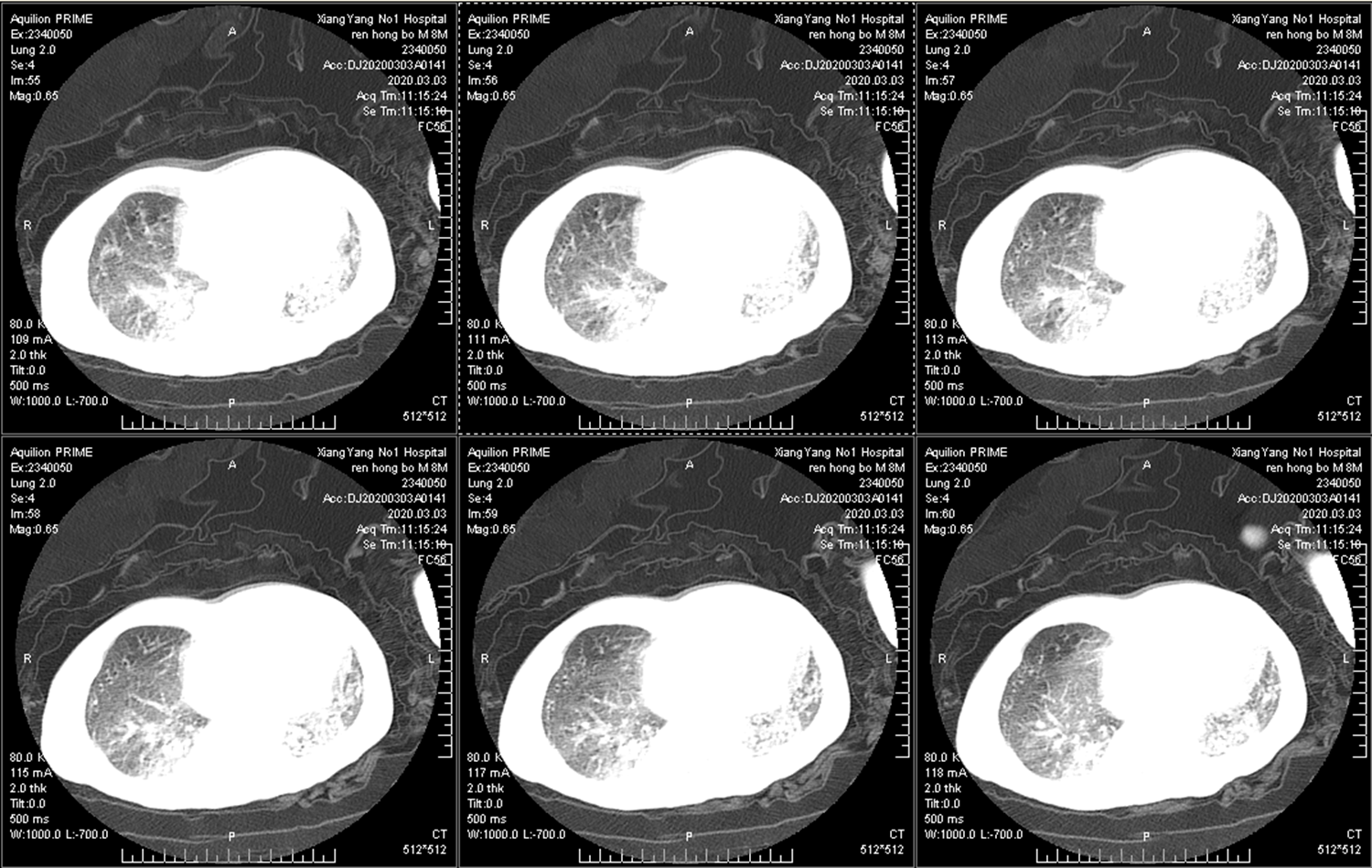


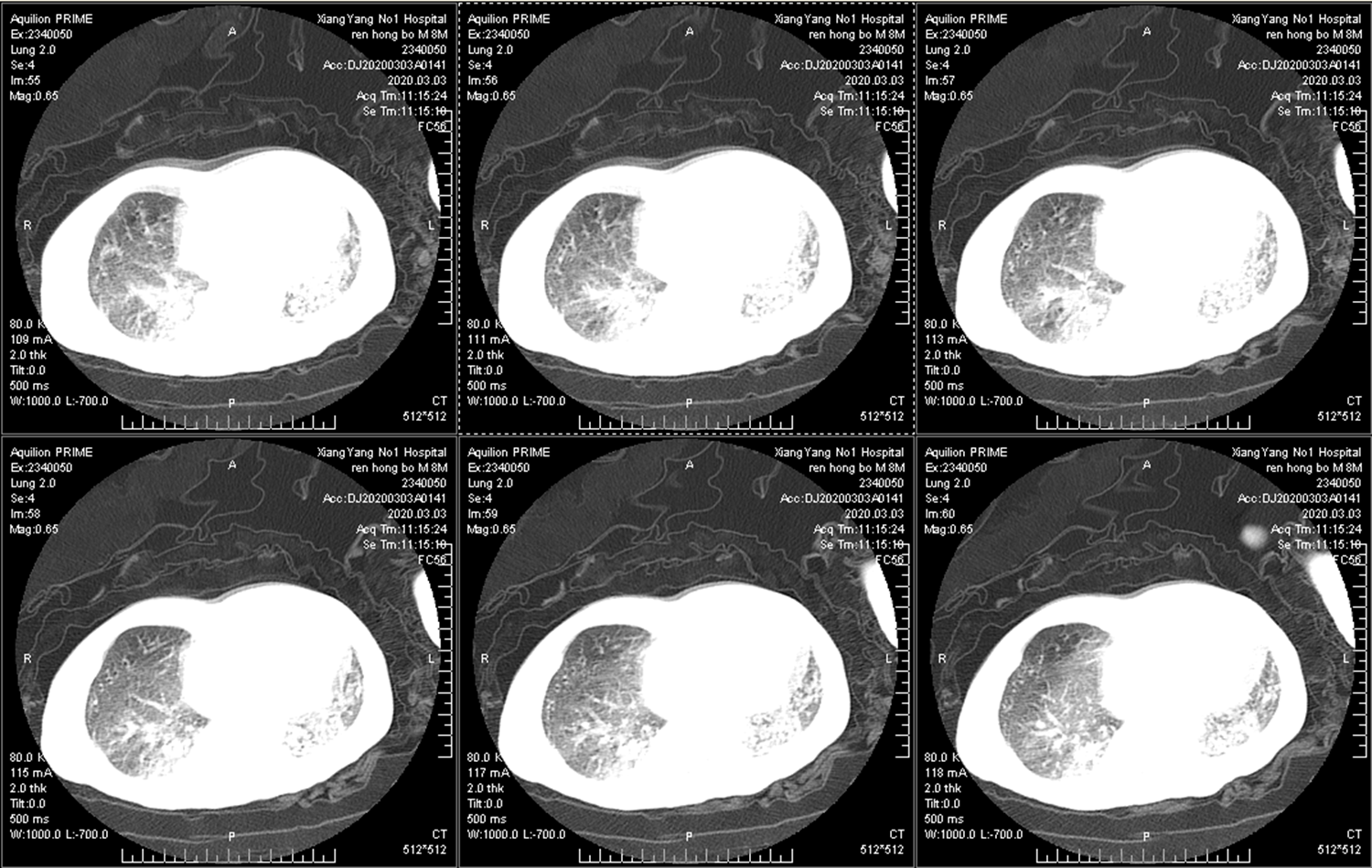


Patchy and flaky high-density shadows in both lungs improved, multiple ground-glass opacity and stripe shadow in both lungs

**Figure S3 Chest CT scans (hospitalization day43, illness day49)**


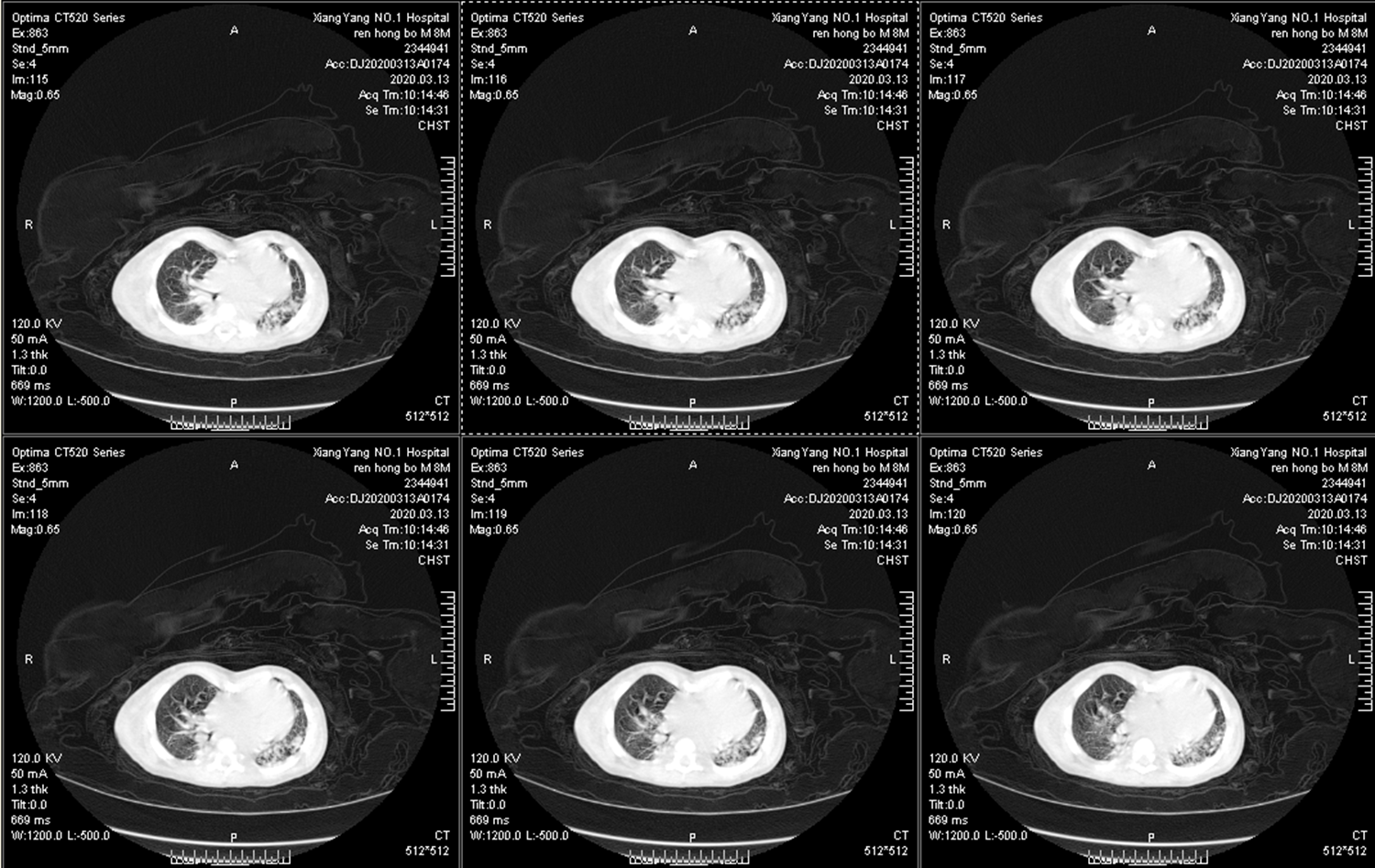


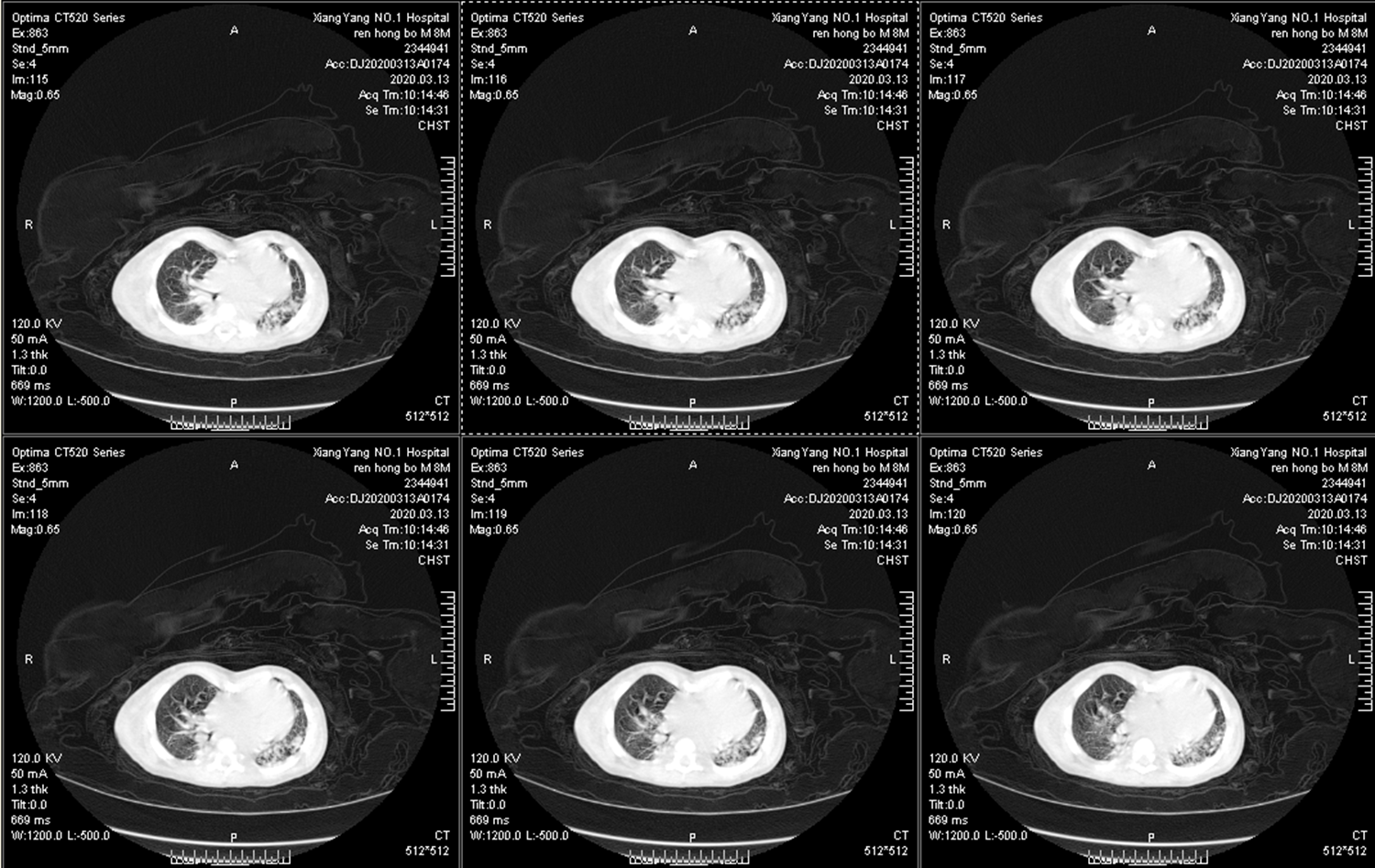


multiple ground-glass opacity and patchy and flaky high-density shadows improved, more stripe shadow in both lungs

**Author Contributions**

Xiaoping Luo conceptualized the study design.Rong Jiao, Aiming Zhang, Liru Qiu decided medical advice, Xi Chen, Fang Zeng, Niannian Tian took care of the infant, Feng Fang,Qin Ning gave the medical advice, Liru Qiu, Rong Jiao collected demographic, clinical, and laboratory data; Yi Zhang, Hao Li did the laboratory tests. Liru Qiu, Rongrong Xu, Menaka Dhuromsingh plotted the figures; Sheng Li, Ziyan Sun interpreted the images of CT scan;Liru Qiu, Yafei Huang, Xiaoping Luo ,Rong Jiao ,Aiming Zhang analyzed the data; Xiaoping Luo,Liru Qiu, Rong Jiao, Feng Fang,Qin Ning,Yu Chen interpreted the results; Liru Qiu wrote the initial drafts of the manuscript; Xiaoping Luo, Menaka Dhuromsingh commented on and revised the manuscript.

All authors read and approved the final report.

**Funding**

Hubei Province Emergency Response Funding #2020FCA006: “Research project on developing a systematic diagnostic and treatment approach for 2019 Novel Coronavirus Pneumonia Disease”

**Acknowledgements:**

We are grateful to the parents of the patient for providing the written informed consent to publish this report.

**Conflicts of interest**

All authors declared no conflicts of interest

**Reference:**

1. Weiss, S. L., M. J. Peters, W. Alhazzani, et al., Surviving Sepsis Campaign International Guidelines for the Management of Septic Shock and Sepsis-Associated Organ Dysfunction in Children. Pediatr Crit Care Med, 2020. **21**(2): p. e52-e106.

2. Khwaja, A., KDIGO clinical practice guidelines for acute kidney injury. Nephron Clin Pract, 2012. **120**(4): p. c179-84.

3. Schwartz, G. J., L. P. Brion, and A. Spitzer, The use of plasma creatinine concentration for estimating glomerular filtration rate in infants, children, and adolescents. Pediatr Clin North Am, 1987. **34**(3): p. 571-90.

4. Pediatrics, Cma Society Of, Recommendations for the diagnosis, prevention and control of the 2019 novel coronavirus infection in children (first interim edition). Zhonghua Er Ke Za Zhi, 2020. **58**.

5. Clinical management of severe acute respiratory infection when novel coronavirus (nCoV) infection is suspected— Interim guidance. Jan,12,2020. world health organization (WHO)
